# Diabetes, Heart Failure, and COVID-19: An Update

**DOI:** 10.3389/fphys.2021.706185

**Published:** 2021-10-15

**Authors:** Carleigh Hebbard, Brooke Lee, Rajesh Katare, Venkata Naga Srikanth Garikipati

**Affiliations:** ^1^Department of Emergency Medicine, The Ohio State University Wexner Medical Center, Columbus, OH, United States; ^2^Department of Physiology–HeartOtago, University of Otago, Dunedin, New Zealand; ^3^Dorothy M. Davis Heart and Lung Research Institute, The Ohio State University Wexner Medical Center, Columbus, OH, United States

**Keywords:** diabetes, heart failure, COVID-19, pandemic, CVD (cardio vascular disease), Long-COVID, SARS-CoV-2, diabetic cardio

## Abstract

The novel severe acute respiratory syndrome coronavirus 2 (SARS-CoV-2) was declared a pandemic by the WHO in March 2020. As of August 2021, more than 220 countries have been affected, accounting for 211,844,613 confirmed cases and 4,432,802 deaths worldwide. A new delta variant wave is sweeping through the globe. While previous reports consistently have demonstrated worse prognoses for patients with existing cardiovascular disease than for those without, new studies are showing a possible link between SARS-CoV-2 infection and an increased incidence of new-onset heart disease and diabetes, regardless of disease severity. If this trend is true, with hundreds of millions infected, the disease burden could portend a potentially troubling increase in heart disease and diabetes in the future. Focusing on heart failure in this review, we discuss the current data at the intersection of COVID, heart failure, and diabetes, from clinical findings to potential mechanisms of how SARS-CoV-2 infection could increase the incidence of those pathologies. Additionally, we posit questions for future research areas regarding the significance for patient care.

## Introduction

**FIGURE 1:**
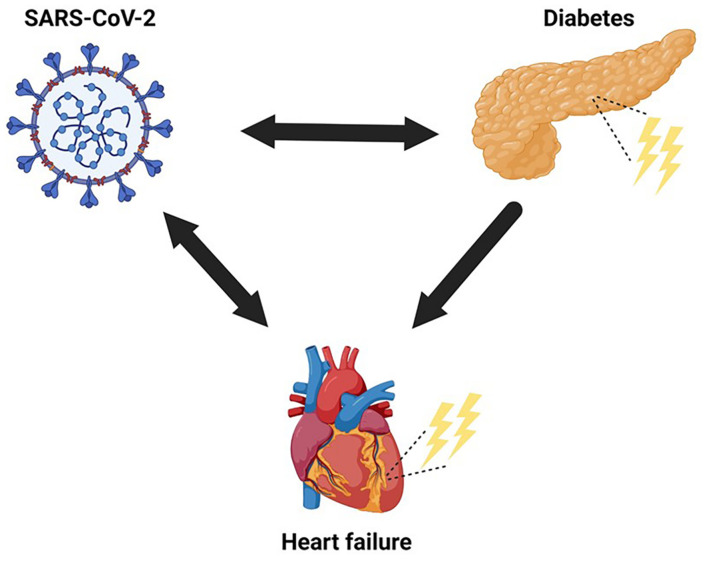
Interconnection between diabetes, heart failure, and SARS-CoV-2.

November 2019 marked the appearance of a novel human infectious RNA virus that has precipitated worldwide crises and left indelible marks on society, science, and healthcare. Named as the second of its kind and for its effects on the human respiratory system, severe acute respiratory syndrome coronavirus (SARS-CoV-2) has, to date, infected over an estimated 200 million persons (Dong et al., 2020). Acute manifestations of the disease range from asymptomatic infection to acute hypoxic respiratory failure and death. While up to 5% of infections result in critical illness (Guan et al., 2020), most acute infections seem to present with few-to-no symptoms (Wu et al., 2020). Though the rate of COVID-19 deaths had declined in the last months throughout most of the world, new variants have appeared, and an accumulating body of epidemiologic and basic science suggests that COVID-19’s aftermath on human health may be longer-lasting than first imagined. We now know that, in addition to causing respiratory illness, SARS-CoV-2 directly and indirectly (and sometimes by unknown methods) can affect multiple organ systems: cardiac, hematologic, pancreatic, renal, and others (Gupta et al., 2020; Siddiqi and Mehra, 2020). Important questions going forward are *what are the long-term effects of SARS-CoV-2 infection; what will be the burden on patients and the healthcare system*; and *how we will continue to best screen and treat patients who have been affected by the disease*. Given the breadth of this topic, in this work, we focus on reviewing the relationships among heart failure, diabetes, and SARS-CoV-2 infection (Figure 1).

A review of the available literature was performed using multiple databases, including PubMed, Google Scholar, bioRxiv, medRxiv, and real-time resources (e.g., WHO reports). Search terms included SARS-CoV-2, Heart failure, diabetic heart failure, mechanisms of infection, long-COVID-19, post-COVID, chronic COVID, post-COVID syndrome, and long-haul COVID, viral illness following COVID-19, post-COVID illness, COVID recovery, predictors of long-COVID-19. Additional literature was found by reading references in those articles as well. Articles and Reviews were curated individually by the authors.

### Current Heart Failure Epidemiology

Heart failure (HF) is a clinical syndrome that carries heavy morbidity and mortality for patients and high healthcare costs for the US health system. In patients over 65 years old, HF exacerbation is a common reason for hospital admission from the Emergency Department, and the risk of death or re-hospitalization in the sixty-to-ninety-day period following the admission is estimated to be as high as 45% (Gheorghiade et al., 2011). The associated yearly healthcare cost of HF in 2012 was estimated to be around $ 40 billion US dollars (Gheorghiade et al., 2011). According to data collected from 2015 to 2018 (Virani et al., 2021), over 6 million Americans have HF and, for the last 11 years, HF prevalence has been on the rise globally (Virani et al., 2021). A silver lining, of course, is that one contributing factor to the increased prevalence is the general increase in life expectancy (with increased age comes increased incidence of HF); However, HF continues to be one of the leading causes of death and fastest-growing categories of heart disease. Pre-COVID data predict a US population prevalence increase from 2.4 to 3.0% by 2030 (Heidenreich et al., 2013).

HF is ubiquitous in the medical setting and, generally, recognizable, contributing to the erroneous impression that “heart failure” is a single entity or disease. Instead, it is a syndrome that can result from different pathophysiologic causes and etiologies and, likely, for that reason, has had a definition that continually undergoes revisions as we discover new information (Bozkurt et al., 2021). However, as analyzed, reviewed, and categorized by Bozkurt et al. (2021), combinations of three fundamental factors unite the various HF presentations: biophysical evidence of cellular and architectural pathophysiology (e.g., elevated brain-natriuretic peptide, fibrosis) (Gjesdal et al., 2011), patient-reported symptomology (e.g., fatigue, shortness of breath, decreased exercise tolerance etc.), and physician-observed signs (e.g., leg swelling, pulmonary edema). This information is pertinent because, given the various current tools we use to look for HF, we have seen signs of heart damage from SARS-CoV-2 with a form of each modality.

**FIGURE 2:**
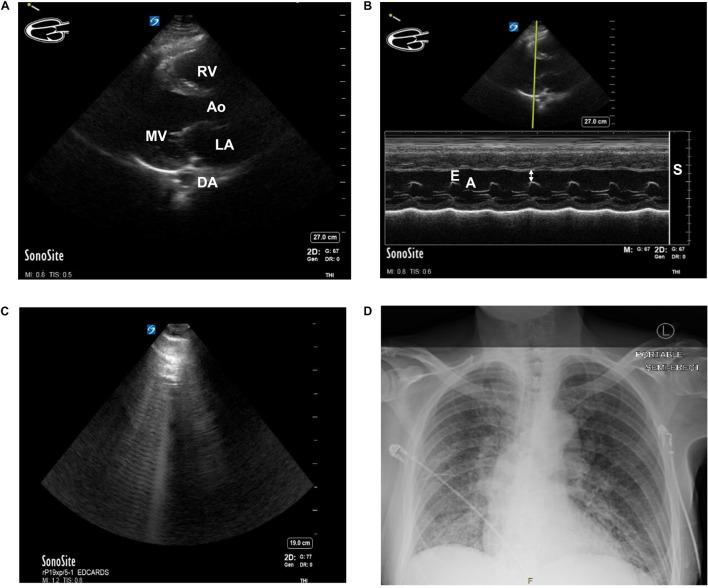
**(A)** Parasternal Long Axis (PLAX) view of the heart of a patient with known heart failure and diabetes using Point of Care Ultrasound (POCUS) imaging technique. MV, mitral valve. Ao, Aortic outflow. DA, descending aorta. LA, left atrium. RV, Right ventricle. **(B)** E-point Septal Separation (EPSS) calculation of the same patient with known heart failure and diabetes using POCUS. M-Mode doppler tracing. Estimated ejection fraction of 32%. Double arrow = EPSS. E = E wave. A = A wave. S = septum. **(C)** B-lines seen while performing cardiac ultrasound in a patient with COVID-19. A patient presented to the Emergency Department in acute hypoxic respiratory failure secondary to SARS-CoV-2 infection. The B-lines pattern seen above is indicative of lung pathology and, in the setting of SARS-CoV-2 infection, often correlates with ground-glass opacities on CT. **(D)** Chest x-ray of the same patient. Findings of bilateral ground-glass airspace disease. With no prior known hypercoagulable risk factors other than age and SARS-CoV-2 infection, this patient was found to have ground-glass opacities on CT and a new pulmonary embolism. Patient consent was obtained for publication and discussion of ultrasound and x-ray imaging.

A standard first-line imaging method that helps delineate structure and function is echocardiography. This method employs sound waves through the chest wall, producing an image as some waves return and others do not. This provides real-time images of the heart beating and allows for calculating both architectural and functional parameters such as muscle thickness and ejection fraction (Figure 2). Magnetic Resonance Imaging (MRI) can provide information about organ structure and give important information about tissue health. Myocarditis, for example, can often be appreciated on MRI. When cardiac tissue or vessels are damaged and/or stressed, specific biomarkers rise. Cardiac troponin, for example, is indicative of myocardiocyte damage and is used with electrocardiogram in standard first-line evaluation of acute myocardial infarction. Brain Natriuretic Peptide (Pro-BNP) is a less reliable protein marker but measures stretch of the vascular system (as might happen if a person were retaining extra fluid from HF).

### Association Between Severe Acute Respiratory Syndrome Coronavirus 2 and Heart Failure

There is growing evidence of a correlation between SARS-CoV-2 infection and myocardial injury (Madjid et al., 2020), even in seemingly healthy individuals. Puntmann et al. (2020), for example, demonstrated through MRI studies that there is an elevated incidence of myocardial injury in people who have recovered from COVID-19, even after controlling for preexisting conditions. Other researchers measured a four-to-five-fold higher increase in poor outcomes for patients with underlying cardiovascular disease (CVD) who are infected with SARS-CoV-2 than for patients without CVD (Li X. et al., 2020; Wu and McGoogan, 2020); and in one United Kingdom study, patients with previously diagnosed CVD had a 50.0% mortality while those without known CVD had only 10.6% (Chatrath et al., 2020). There are even questions as to what long-term cardiac damage the virus might cause to the healthiest of individuals: Rajpal et al. (2020), found evidence of active myocarditis in 15% of young athletes who had tested positive for SARS-CoV-2 infection after 11–53 days of quarantine, raising general questions about recovery times from the virus (discussed later).

#### Evidence of Global Cardiac Dysfunction After Severe Acute Respiratory Syndrome Coronavirus 2 Infection

Various clinical data modalities show mixed support for the epidemiologic and statistical findings that SARS-CoV-2 infection could be causing or exacerbating myocardial damage, possibly permanently in some cases. In a prospective echocardiography study of 100 patients admitted to the hospital with COVID-19, 69% showed signs of heart failure. Among these, 39% had right ventricular (RV) dilation or dysfunction, 16% had left ventricular (LV) diastolic dysfunction, and 10% had LV systolic dysfunction (Szekely et al., 2020). Areas of significant difference between patients who developed echocardiographic signs of heart failure included admission levels of creatinine (a marker of kidney function and/or perfusion), pro-BNP, systolic blood pressure, and C-reactive protein (a marker of inflammation and important topic discussed later). A retrospective study from New York on 110 patients with COVID-19 (Argulian et al., 2020) also identified RV dilation in 31% of patients who underwent echocardiography. The patients also showed a significant impairment in kidney function. As noted by Szekely et al. (2020) the likeliest explanation for the preponderance of right-heart failure in these patients is an acute increase in pulmonary resistance secondary to multiple mechanisms involved during COVID-19 (Figure 2), though, in a paper by Graziani et al. (2020), patients with COPD had higher mortality with COVID than those without but a similar incidence of HF. When patients were stratified by worsening clinical grade, there was not a difference in the LV dysfunction seen (Szekely et al., 2020). An interesting comparison might be a retrospective cohort of case-controls (prior to 2019) with echocardiographic RV and LV data.

#### Evidence of Tissue Cardiac Damage After Severe Acute Respiratory Syndrome Coronavirus 2 Infection

Cardiac magnetic resonance imaging (CMR) techniques combined with serum biomarkers have identified cardiac tissue damage in patients recovering from COVID-19 infection. Puntmann et al. (2020) produced a prospective cohort study of 100 German patients who had tested positive for SARS-CoV-2 infection but were symptomatically recovered and subsequently had tested negative since their infection. Of 100 patients considered “recovered” from SARS-CoV-2, 78% had abnormal CMR images consistent with myocardial inflammation, heart scarring, or pericardial abnormalities, and this was true even after controlling for preexisting conditions. Additionally, there was a significant increase in serum troponin levels in patients who recovered from infection when compared to persons who never had SARS-CoV-2 infection. Similar to Argulian et al. (2020) and Szekely et al. (2020), Puntmann et al. (2020) found some RV dysfunction in some of these patients. Dissimilarly, however, Puntmann et al. (2020) did find evidence that patients also had lower left ventricular ejection fraction and higher left ventricle volumes when compared to controls. Particularly salient details of this work are that the authors excluded from the study any patients who were being worked up by their doctors for cardiac disease, which means there is a group of individuals who symptomatically really have not yet recovered from infection despite subsequent negative tests. Sobering, too, is that 67% of the patients studied had mild/moderate-to-no symptoms. In another study, 26 Ohio State University (OSU) athletes who tested positive for SARS-CoV-2 infection did not show any elevation in cardiac biomarkers, yet CMR imaging showed evidence of myocarditis in 15% of the athletes and cardiac scarring in 30% (though, unknown if that scarring is COVID-related or exercise-related change) (Rajpal et al., 2020). Many of the athletes were asymptomatic and it would be interesting to know how this compared to hearts from athletes who had not contracted SARS-CoV-2. In a meta-analysis of studies from the United States, Asia, Europe, and Brazil, Toraih et al. (2020) found elevated levels of Troponin I and NT-proBNP and others have reported similar findings (Shi et al., 2020; Zhou et al., 2020).

#### Clinical Signs and Symptoms of Possible Heart Dysfunction After Severe Acute Respiratory Syndrome Coronavirus 2 Infection

We now know that some people who have had a SARS-CoV-2 infection have long-persisting symptoms—such as fatigue and dyspnea—or fail to recover from the infection entirely. “Long-COVID” was noticed anecdotally first by individuals communicating in blogs and Social Media but since has been corroborated by large data sets from phone applications, and the NIH recently have launched initiatives to study the biological causes of this phenomenon [as reviewed by Nath (2020), Sudre et al. (2021)]. It may be that Long-COVID is a form of post-sepsis syndrome (Prescott and Girard, 2020; Gritte et al., 2021), and, if so, we might expect increased re-hospitalization of these patients who have prior comorbidities. It will be interesting to study these individuals’ demographics, risk factors, biomarkers, and cardiac imaging and evaluate whether there might be lingering myocarditis or even pre-heart failure.

Though question-provoking, these results of cardiac abnormalities may not be surprising. During the 2002–2004 SARS outbreak, researchers discovered increases in patients’ cardiovascular complications following SARS-CoV-1 infection (Peiris et al., 2003; Chong et al., 2004). To date, the SARS-CoV-2 virus has had a wider reach than the SARS-CoV-1 virus, infecting an estimated population total of over one-hundred million people (worldmeters, 2021). It is unclear if SARS-CoV-2 infection is unmasking underlying heart failure and/or causing direct myocardial damage, and, if the latter, if that damage will have permanent long-term health effects for the individual. *Does cardiac involvement occur and persist in people with mild to moderate COVID-19 infections, even after recovery from symptoms?* No matter the answer to these questions, we need to consider and anticipate what, if any, consequences SARS-CoV-2 infection may bring to morbidity and mortality for patients and cost to healthcare systems.

### Association Between Severe Acute Respiratory Syndrome Coronavirus 2 and Diabetes

There appears to be an increase in diabetes incidence in patients who have been infected with SARS-CoV-2 (Al-Aly et al., 2021). Though an increase in power would be needed before definitive conclusions are drawn, there are multiple reports of increases in previously undiagnosed type I diabetes in adults (Accili, 2021) and children (Unsworth et al., 2020) during the COVID-19 pandemic. In one United Kingdom study of a few hospital units, a significantly higher increase in new-onset type I diabetes was observed in children aged 23 months to 16.8 years of age (Unsworth et al., 2020). In a study from China (Li J. et al., 2020), patients with COVID-19, with or without previously diagnosed diabetes, had a higher prevalence of ketosis and ketoacidosis. Acute illness is one of the most common precipitating factors of new diagnoses of type I diabetes, a characteristic way in which diabetes first might be discovered in children or adolescents. Despite SARS-CoV-2 causing reportedly mild symptoms in children compared to adults, one might reasonably hypothesize that a virus with high infectivity and infection incidence naturally could cause a concomitant increase in type I diabetes incidence and prevalence in predisposed individuals. There is, however, also a well-documented but less understood relationship between hyperglycemia and infection with coronaviruses, with or without diabetes, and an increased incidence of mortality. A few proposed mechanisms include viral binding to ACE2 receptors—receptors found in nasopharyngeal tissue, pancreatic cells, and others. More on proposed mechanisms will follow. Additionally, in a study of thousands of adult patients from the US Department of Veterans Affairs, Al-Aly et al. (2021) did find an increase in diabetes and insulin prescriptions. As the authors note, it is difficult to explain the root cause of the increase (inactivity during COVID quarantine, better follow up direct infection of the virus causing diabetes, etc.). What we do know, though, is that having diabetes does increase a person’s risk of having heart failure.

### Diabetic Heart Disease

DHD is defined as the presence of heart disease specifically in patients with diabetes that encompasses coronary artery disease, heart failure, and/or cardiomyopathy (Marwick, 2008; Lew et al., 2017). DHD is a broad definition that encapsulates many myocardial diseases due to the varying etiology and the poorly understood mechanisms. Therefore, DHD should be considered as a distinct clinical entity and not limited to one particular type of myocardial disease (Lew et al., 2017). A recent systemic literature review conducted pre-covid era identified that CVD affects approximately 32.2% of individuals with type 2 diabetes, and, significantly, 14.9% of them developed HF. Of note, CVD was the cause of death in 9.9% of individuals with type 2 diabetes, representing 50.3% of all deaths, demonstrating that diabetes is an independent risk factor for CVD and associated mortality (Einarson et al., 2018). This is only expected to increase, especially with the incidence of type 2 diabetes affecting over 592 million people globally by 2035, a sharp increase from 382 million in 2013 (Guariguata et al., 2014).

The underlying mechanisms leading to DHD development remain unclear, although increasing evidence suggests that hyperglycemia and insulin resistance lead to DHD development. Hyperglycemia activates the polyol pathway, protein kinase C, advanced glycation end products, and hexosamine pathway, while insulin resistance activates Ras/MAPK pathway. These induce myocardial lipotoxicity and augment oxidative stress and systemic inflammation, resulting in endothelial cell dysfunction, cardiac hypertrophy, fibrotic scarring, and apoptotic cell death, thereby compromising cardiac function. Interestingly, the clinical treatment of DHD is solely dependent on a cocktail of medications and symptomatic treatment approaches. There is no single drug that specifically and effectively treats/prevents DHD. This is likely due to the multifactorial nature of the underlying mechanisms.

Individuals with diabetes have more than two times the risk of developing HF compared to non-diabetic individuals. The Framingham Heart Study suggested diabetes independently increases the risk of HF up to two-fold in men and five-fold in women even after adjusting for other risk factors such as age, hypertension, hypercholesterolemia, and coronary artery disease (Kannel, 1979). While an association of diabetes in HF with reduced ejection fraction (HFrEF) is well established, recent evidence suggests heart failure with preserved ejection fraction (HFpEF) is a common comorbidity of diabetes (Solomon et al., 2018), with a prevalence of almost 45% (Echouffo-Tcheugui et al., 2016). Furthermore, outcomes following HFpEF are poor and comparable to HFrEF, and sudden death accounts for around 20% of mortality in persons with diabetes who also have HFpEF (Vaduganathan et al., 2018).

Adding to the insult, patients with heart failure may be particularly susceptible to COVID-19 complications. Along these lines, recent reports suggest mortality rates were significantly higher for patients with HF with COVID-19 across broad cohorts, including those with active cancer on chemotherapy. However, mortality attenuated due to increased testing, disease-modifying therapy, and improved COVID-19 care. Despite all these, patients with underlying HF who contract COVID remain at high-risk for in-patient mortality (Panhwar et al., 2019; Alvarez-Garcia et al., 2020; Chatrath et al., 2020; Lee L. Y. et al., 2020; Bhatt et al., 2021; Group et al., 2021).

### There Is an Increased Risk of Heart Failure in Patients With Diabetes Who Contract COVID-19

Available evidence suggests that COVID-19 has poor outcomes for patients with diabetes (Table 1) and is likely to exacerbate heart failure in individuals with diabetes (Freaney et al., 2020). Patients with diabetes and CVD have a higher propensity for severe outcomes when infected with SARS-CoV-2 than those with diabetes or heart failure alone. In a meta-analysis of six studies with > 1,500 patients (Li B. et al., 2020), cardiac-cerebrovascular disease and diabetes were present in 16.5 and 9.7% of the patient population. In patients in the ICU or with severe cases, the prevalence of cardio-cerebrovascular disease and diabetes was present at three- and two-fold higher rates. In a large United Kingdom study (61,414,470 individuals) on COVID-19 mortality (23,698 deaths), 30.9% of individuals who died had preexisting coronary artery disease, and 17.8% had heart failure. 31.4% of individuals who died had type 2 diabetes, 1.5% had type 1 diabetes, and 0.3% had other types of diabetes (Barron et al., 2020). It is estimated to be about five times greater than the percentage of diabetes in the United Kingdom. Notably, individuals with type 1 and type 2 diabetes who died were younger than those without diabetes.

**TABLE 1:** Characteristics COVID-19 patient’s grouped number of patients admitted, gender, age,% diabetes,% CVD and% mortality.

Study name	Date	# of COVID positive patients admitted	Percent Male	Percent Female	Median Age	Mean Age	Percent Diabetes	Percent CVD	Percent. Mortality
Wang G. et al. (2020)	Mar-20	242	49.20	50.80	45		6.20	3.70	0.80
Qi et al. (2020)	Mar-20	267	55.80	44.20	48		9.70	4.90	1.50
Cao et al. (2020)	Mar-20	198	51.00	49.00			7.60	6.00	NA
Liu S. et al. (2020)	Mar-20	620	52.60	47.40			6.50	2.60	NA
Chen M. et al. (2020)	Mar-20	123	49.60	50.40	53 (discharged) 72 (death)		11.40	16.00	25.20
Chen X. et al. (2020)	Mar-20	291	49.80	50.20	46		7.60	4.10	0.70
Hong et al. (2020)	Mar-20	140	51.00	49.00		45.66	9.00	3.00	NA
Wang S. et al. (2020)	Apr-20	165	55.80	44.20	44		8.20	4.80	0.60
Zheng et al. (2020)	Apr-20	30	43.30	56.70	44.5		10.00	3.30	NA
Yuan et al. (2020)	Mar-20	417	47.50	52.50		45.40	7.70	6.70	0.72
Petrilli et al. (2020)	Mar-20	1,999	62.60	37.40	62		25.20	44.60	14.60
Guan et al. (2020)	Mar-20	1,099	58.10	41.90	47		7.40	2.50	1.40
Bai et al. (2020)	Dec-20	1,833	66.10	33.90	70		19.50	17.90	100.00

Recently, Abe et al. (2021) showed that patients with diabetes admitted with COVID-19 had increased incidence of acute myocarditis, acute heart failure, acute myocardial infarction, and new-onset atrial fibrillation. They concluded that diabetes was associated with worse cardiovascular outcomes. Furthermore, COVID-19 appears to increase the risk of HFpEF, which, as described above, is beginning to be recognized as one of the major forms of HF (Freaney et al., 2020). Since HFpEF is high in individuals who have diabetes, there may be a positive relationship between COVID-19, HFpEF, and diabetes. While no direct data support this notion yet, indirectly, the central pathogenesis shared by HFpEF and COVID-19 appears to be inflammation. Infection with SARS-CoV-2 increases the release of pro-inflammatory cytokines such as IL-1 and IL-6, which directly affect both the respiratory system and myocardium (Freaney et al., 2020). Therefore, when an individual with diabetes and HFpEF contracts COVID-19, it is likely to exacerbate the pathology of HFpEF in these patients. In addition to inflammation, obesity is a significant risk factor for COVID-19 severity. Further, more than 20% of patients with diabetes struggle with obesity and obesity is also a risk factor of HFpEF. Combined, inflammation and obesity could increase the risk of HF in diabetic patients with COVID-19.

Taken together, available evidence suggests that COVID-19 may induce myocardial damage and HF and, significantly, is particularly detrimental to individuals with diabetes. Further, COVID-19 may directly induce diabetes. Some propose that there is perhaps a common mechanism by which the virus worsens diabetes and HF concomitantly and synergistically, and the next section will review the main potential mechanisms.

## Mechanisms

### The Virus

**FIGURE 3:**
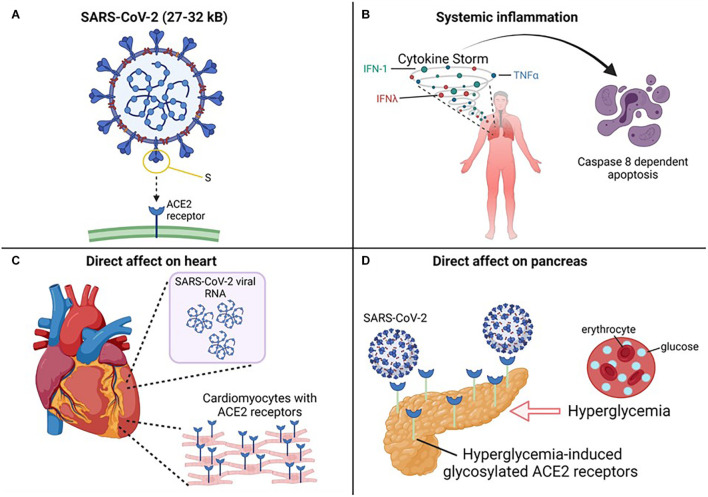
Molecular mechanisms of SARS-CoV-2 infection.

Understanding the structure and targets of coronaviruses helps frame the mechanism of the virus’s attack and some of the pathophysiology caused by the virus’s aftermath (Figure 3). All coronaviruses are positive-sense, single-strand RNA-based viruses with crown-like spikes. This *crown* of spikes is what gives the virus its name. Coronaviruses share the same overall structure, its genome - SARS-CoV-2’s being large for RNA viruses (ranging from 27 to 32 kb) – is found inside a capsid formed by the nucleocapsid protein and surrounded by an envelope. Three proteins are attached to the envelope: the membrane protein and envelope protein are involved in virus assembly, whereas the spike protein (S) is responsible for viral attachment and entry. The spike protein comprises S1, which includes the receptor-binding domain (RBD), and S2, which allows the fusion between the virus and the host membranes (Li, 2016).

While the coronavirus family is quite large, SARS-CoV, SARS-CoV-2, and MERS belong to the same genus, Betacoronavirus. Even though these viruses share clinical similarities, there are critical differences among them. SARS-CoV-2 shares 79% sequence similarity to SARS-CoV and only 50% to MERS (Lu et al., 2020). SARS-CoV-2 has higher infectivity but lower mortality (<2%) compared to MERS (∼34%) and SARS-CoV (∼9%). MERS bind to the dipeptidyl peptidase (DPP4) receptor, while SARS-CoV-2 and SARS-CoV bind to the ACE2 receptor (Petrosillo et al., 2020). Even in binding to ACE2, key differences have been observed where the differences in the mechanism are possibly speculated to help explain the difference in infectivity between SARS-Cov2 and SARS-CoV (Shang et al., 2020; Ozono et al., 2021). Even considering these significant differences, the long-lasting effects on people infected with these viruses share similarities (Li et al., 2003; Peiris et al., 2003; Hui, 2005; Ahmed et al., 2020; Chasouraki et al., 2020; Xiong et al., 2020) which are worth investigating to understand if there is a common underlying mechanism. Researchers have proposed a few potential mechanisms of how SARS-CoV2 is involved in causing severe outcomes for people with cardiovascular disease and/or diabetes mellitus (Figure 3). These are discussed below.

### Systemic Inflammation

Systemic inflammation is one of the most common findings in autopsies of patients who had COVID-19. Findings include focal pancreatitis, myocardial inflammation, and others (Eketunde et al., 2020). Researchers also observed inflammation in the brain (Lee L. Y. et al., 2020). While virus infections are known to cause direct damage to heart cells, Lee M. H. et al. (2020) concluded the brain inflammation likely was caused through an indirect mechanism secondary to the systemic response to SARS-CoV-2 infection. Providing support for this hypothesis, Hadjadj et al. (2020) observed an impaired interferon type I response in patients with COVID-19 with a high viral load and heightened inflammatory response. Clinically, inflammation and immune response are often two-edged swords, necessary to help the body fight and apparent infection, but sometimes resulting in damage to organs.

The acute hypoxic respiratory failure and ARDS-like picture seen in patients with severe COVID-19 is thought to be triggered by a cytokine storm (Hojyo et al., 2020). Additionally, cytokine storm has been put forward as a mechanism contributing to left ventricular dysfunction in patients with SARS (Li et al., 2003). Therefore, it is possible that hyper-inflammation and a modulated immune response could also explain cardiovascular damage in COVID-19 patients. In a study of 217 patients, IL-6 and lactate dehydrogenase were detected within 24 h of hospital admittance (Zeng et al., 2020). Because IL-6 has pro-inflammatory properties, this has been proposed as one factor responsible for severe diabetes mellitus and COVID-19 infection (Lim et al., 2021). However, recent clinical trials targeting IL-6 with tocilizumab or sarilumab have shown mixed results (Stone et al., 2020; Cavalli et al., 2021; Hermine et al., 2021; Parr, 2021; Salvarani et al., 2021). Cavalli et al. further showed that inhibiting IL-1 with anakinra significantly reduced mortality risk compared to inhibiting IL-6. In this study, IL-6 inhibition seemed to be only effective in patients either with high C-reactive protein or lactate dehydrogenase concentrations (Cavalli et al., 2021). In another study, multiple cytokines were detected in COVID-19 patients with mild or moderate symptoms, namely IL-1α, IL-1β, IL-17A, IL-12 p70, and IFNα (Yale et al., 2020). In the same study, patients with severe symptoms also were shown to have IL-33, IL-16, IL-21, IL-23, IFN-λ in their blood, as well as thrombopoietin (TPO) eotaxin, and eotaxin 3 (Yale et al., 2020).

Recently, researchers studying COVID-19 cytokine storm in bone marrow-derived macrophages (BMDMs) have proposed a possible mechanism linked to programmed cell death that can explain systemic inflammation not only in COVID-19 but also potentially in MERS, SARS, and other viruses where cytokine storm has been observed (Channappanavar and Perlman, 2017; Karki et al., 2021; Ryabkova et al., 2021). The researchers demonstrated cell death only when both TNF-α and IFN-λ were applied as a treatment, and not in any other combination of cytokines, individually or in combinations that did not include TNF-α and IFN-λ; furthermore, in their analysis of data from Silvin et al. (2020), they observed that while TNF-α production peaked in patients with moderate disease, IFN-λ only peaks in patients with severe COVID-19. This observation is also supported by RNA-seq data that suggests the increase in production of TNF-α and IFN-λ is driven by immune cells (Lee J. S. et al., 2020). The researchers also showed that a combination of TNF-α and IFN-λ production causes inflammatory cell death *via* IRF1/STAT1 pathway expression. IRF1/STAT1 expression results in the production of iNOS and NO, which ultimately activates caspase-8 dependent apoptosis. This programmed cell death was demonstrated not to be caused by suppression of NF-κB, nor was it due to intrinsic apoptosis in macrophages.

### Direct Injury

Viral particles have been found within cardiomyocytes in patients infected with SARS-CoV-2 (Dolhnikoff et al., 2020; Tavazzi et al., 2020). Viral RNA of SARS-CoV-2 has also been detected in cardiac tissue during autopsies (Lindner et al., 2020; Liu P. P. et al., 2020). This is similar to what was observed in SARS-CoV-1 infection as well (Oudit et al., 2009). Researchers have also observed *in vitro* direct infection by SARS-CoV-2 in human iPSC-derived cardiac cells (Pérez-Bermejo et al., 2020). In this study, the researchers observed cytopathic damage by SARS-Cov-2, resulting in myofibrillar fragmentation and nuclear DNA loss in intact cells, even if the virus was not actively replicating. The same researchers also observed similar cytopathic results in myocardium cells of patients with COVID-19. These data suggest that SARS-CoV-2 can directly infect cardiac cells, and the prevailing hypothesis of SARS-CoV-2 entry into cells is through the ACE2 receptor (Liu P. P. et al., 2020). A recent study showed that the virus could gain entry into a human cell line *via* the ACE2 receptor (Hoffmann et al., 2020). In a protein profiling study using immunohistochemistry, researchers profiled over 150 proteins and found ACE2 was significantly expressed in cardiomyocytes and renal tubules (Hikmet et al., 2020). While ACE2 was detected by both immunohistochemistry and transcriptomics in the pancreas, expression was not as high as in the heart or kidneys (Hikmet et al., 2020).

It is conceivable that direct injury mediated through ACE2 could explain a link between heart failure and diabetes mellitus in COVID-19 patients, which would explain how the virus could be directly involved in damaging the heart and pancreas. However, in a recent analysis of transcriptomic data, the presence of ACE2 in the pancreatic islet β cells were not conclusive: TMPRSS2 was not co-expressed with ACE2 in these cells, which is a necessary co-factor to facilitate SARS-CoV-2 cellular entry (Coate et al., 2020; Hoffmann et al., 2020; Ozono et al., 2021); however, with SARS, diabetes was in some cases was shown to persist for 3 years after infection, indicating potential damage to islet β cells (Yang et al., 2010).

#### Role of Renin–Angiotensin–Aldosterone System

ACE2 is part of the Renin–angiotensin–Aldosterone System (RAAS) system, and its role in potential direct injury by the virus has been discussed above. While ACE is involved in causing a cascade that results in vasoconstriction and inflammation *via* converting angiotensin I to angiotensin II, ACE2 breaks down angiotensin II and thus simultaneously decreases the afterload on the heart (*via* vasodilation) and promotes glucose tolerance (Chappel and Ferrario, 2006; Chhabra et al., 2013). Thus, ACE2 may help protect the cardiovascular system from the remodeling that occurs secondary to increased afterload. Previously, SARS-CoV-1 was shown to reduce ACE2 expression (Kuba et al., 2005), leading to cardiac injury. Perhaps SARS-CoV-2 affects the RAAS system similarly (Luo et al., 2021), which could explain a link between diabetes and cardiac injury seen in COVID-19 patients. This protective effect of ACE2 seems contradictory to its role in virus entry in cells. This contradiction can be explained by hyperglycemia-induced glycosylation (discussed below).

### Hyperglycemia

Hyperglycemia has frequently been found in COVID-19 patients with severe outcomes (Bode et al., 2020). Interestingly, a history of diabetes and hyperglycemia was also found to result in severe outcomes with patients infected with SARS (Yang et al., 2006); thus, a proposed mechanism for hyperglycemia in SARS involved the hyperglycemia-induced glycosylation of ACE2 and the viral spike protein, potentially modulating the binding ability of the virus to the receptor. A similar hypothesis has been proposed to SARS-CoV-2 as well (Brufsky, 2020).

Brufsky notes a discrepancy in gene expression in recent survey studies where ACE2 expression was an inverse correlation of ACE2 expression to disease severity (Chen J. et al., 2020). This discrepancy can be explained by protein glycosylation, a posttranslational modification that gene expression cannot detect. This would suggest that the glycosylated ACE2 receptor is responsible for virus binding and fusion while still explaining the benefit provided by non-glycosylated ACE2.

## Conclusion and Future Directions

In this work we reviewed recent reports supporting that SARS-CoV-2 infection was directly and indirectly associated with adverse health outcomes among all people, but especially for those with HF and/or diabetes. There is a link among diabetes and heart failure and SARS-CoV-2 infection that stretches beyond mere pre-infection comorbidity (Figure 1) and has the potential to have long-lasting effects on the global health system. Just a small increase in the incidence of diabetes or cardiac dysfunction could have profound consequences in overall health burden and number of required screening exams. Currently, some members of the European Society of Cardiology posit that we reasonably might screen (or continue to monitor) for cardiac dysfunction in (1) patients who had demonstrated cardiac dysfunction during their COVID infection; (2) patients with clinical symptoms of dyspnea, exercise intolerance, long COVID symptoms; (3) anyone who had elevated cardiac troponin levels during infection (Richter et al., 2021). Arguments for screening the latter group seem supported by the evidence in Puntmann et al. (2020). Puntmann et al. (2020) and Rajpal et al. (2020) also noted tissue-level signs of cardiac stress in a large percentage of asymptomatic or mildly symptomatic individuals.

We find more questions than answers with the available data, especially regarding patient care and screening in the post-COVID era. *In the acute setting, when the treatment for one aspect of the disease can exacerbate another (such as glucocorticoids dampening inflammation but causing hyperglycemia), can we optimize treatments for diabetes and heart failure using the familiarity with the patient, understanding of pharmaco-kinetics/-dynamics in illness, and consideration of the practicalities in practicing medicine (Bornstein et al., 2020; Hartmann-Boyce et al., 2020; Korytkowski et al., 2020; Solerte et al., 2020; Dagan et al., 2021). Is there a role for TNF-α and IFN-λ inhibition (Lee J. S. et al., 2020)?* There is growing acceptance of and interest in the chronic, symptomatic long-term effects of COVID-19, or “Long-COVID” with multiple trials and studies initiated. *What is “Long-COVID” and is it similar to or a form of post-sepsis syndrome, and does it have cardiac components? Is it possible that a common mechanism explains the multi-organ effects and links SARS and MERS with COVID-19? Are we seeing a real increase in diabetes incidence (Al-Aly et al., 2021), and if so, what is the root cause and type of diabetes? Could it be direct or indirect damage to pancreatic cells, increased detection from increased healthcare screening for the disease, ramifications of inactivity during quarantine, or something entirely different?* The answers to these and like questions are important because the answers dictate treatment and guide screening. Primary care physicians and bodies such as the US Preventive Services Task Force, who help create evidence-based guidelines for physicians caring for patients, will undoubtably be monitoring the developments. Importantly, too, *what is the true incidence of infection and what are the long-term sequelae for asymptomatic individuals?* We saw the preliminary data in one study that 67% of mildly symptomatic to asymptomatic patients still have physiologic evidence of disease damage post-infection (Puntmann et al., 2020). Are they at risk for developing HF or diabetes earlier than before or at all? That answer might help us to calculate the cost-benefit to screening for SARS-CoV-2 antibodies, or prior infection. With more data, we may begin to answer some of these questions.

Armed with new and old knowledge about potential coronavirus infection mechanisms, we may begin to discover new ways to optimize old treatments in the setting of coronavirus infection or even develop entirely new preventative measures. As the fight continues, we look forward to progress in science and medicine.

## Author Contributions

CH, BL, and RK collected the material and wrote the manuscript. VNSG provided critical feedback and helped to shape the manuscript. All authors contributed to the article and approved the submitted version.

## Conflict of Interest

The authors declare that the research was conducted in the absence of any commercial or financial relationships that could be construed as a potential conflict of interest.

## Publisher’s Note

All claims expressed in this article are solely those of the authors and do not necessarily represent those of their affiliated organizations, or those of the publisher, the editors and the reviewers. Any product that may be evaluated in this article, or claim that may be made by its manufacturer, is not guaranteed or endorsed by the publisher.
